# Keap1-Inhibitory Peptides from *Ganoderma lucidum* Spores: Virtual Enzymolysis, Fragmentomics and Antioxidant Mechanism

**DOI:** 10.3390/molecules31122157

**Published:** 2026-06-18

**Authors:** Beibei Chen, Liang He, Qi Huang, Yanbin Wang

**Affiliations:** 1 Key Laboratory of Biological and Chemical Utilization of Zhejiang Forest Resources, Zhejiang Academy of Forestry, Hangzhou 310023, China; chenbeib957@163.com (B.C.); heliang@zjforestry.ac.cn (L.H.); 2 School of Biological and Chemical Engineering, Zhejiang University of Science and Technology, Hangzhou 310023, China; huangqi@zust.edu.cn

**Keywords:** virtual enzymatic hydrolysis, simulation screening, antioxidant peptides, molecular docking

## Abstract

*Ganoderma lucidum* spores protein (GLSP) holds significant potential for providing antioxidant peptides. We employed in silico enzymatic hydrolysis to generate small peptide fragments by specific proteins. Through fast computer screening and molecular docking with Keap1 receptor, we identified two potential antioxidant peptides, KAF (Lys-Ala-Phe) and NDSF (Asn-Asp-Ser-Phe), from 1171 candidates after efficient hydrolysis by pepsin and proteinase K. Molecular docking result showed both of them could bind onto the Leu557, Ala 510 and Val512 of bioactive pockets of Keap1 through hydrogen bonds and NDSF had lower docking energy (−85.6073 kcal/mol). The in vitro antioxidant validation indicated both of them could eliminate DPPH and ABTS radicals dramatically, and NDSF had a stronger scavenging capacity on DPPH (IC_50_ = 35.1 μg/mL) and ABTS (IC_50_ = 55.9 μg/mL), respectively. Quantitative chemical analysis further revealed that the key antioxidant active sites of NDSF were located at O18 of Ser amino side chain, and N9 of Lys terminal amino residue for KAF. Furthermore, in the cellular experiments, NDSF and KAF effectively increased the activities of antioxidant enzymes such as SOD, CAT, and GPx, while also reducing the level of MDA. Together, these findings highlight the potential of *Ganoderma lucidum* spore proteins as a source for the rapid identification of antioxidant peptides. The two selected peptides, therefore, s hold promising prospects for applications in functional foods and health products.

## 1. Introduction

Under normal physiological conditions, cells metabolize reactive oxygen species (ROS), and the accumulation of ROS within cells does not affect the cells but has specific biological functions [1]. When the body is stimulated by external factors, the imbalance between the accumulation of ROS and the endogenous antioxidant system of cells leads to oxidative stress (OS) [2]. Under such circumstances, excessive ROS may be opt to oxidize and modify proteins, lipids, and nucleic acids, triggering inflammatory responses and, in severe cases, causing necrotic cell death [3,4], and developing various diseases such as atherosclerosis, chronic obstructive pulmonary disease, type 2 diabetes, and various cardiovascular diseases, which are often associated with oxidative stress [5].

A large number of studies have shown that targeting the Kelch-like ECH-associated protein 1 (Keap1)-nuclear factor erythroid 2-related factor (Nrf2)-antioxidant response element (ARE) signaling pathway may play a protective role in various oxidative stress and inflammation-related diseases [6]. Under normal conditions, Keap1 binds to the ETGE sequence of Nrf2 through its Kelch domain. Nrf2, via two motifs in its Neh2 domain—the high-affinity ETGE motif and the low-affinity DLG motif—interacts with the Kelch domain of Keap1, which leads to Nrf2 being anchored and subsequently degraded [7]. The disruption of the interaction between Keap1 and Nrf2 has been proven to be a promoting mechanism for the release and translocation of Nrf2 into the nucleus, thereby activating downstream antioxidant response elements [8]. Therefore, an effective strategy to enhance the body’s antioxidant capacity involves developing bioactive compounds that can competitively bind to Keap1 and Nrf2. Currently, the development of Keap1 inhibitors, whether they are chemical drugs or components such as carbohydrates or flavonoids derived from natural products, has been extensively explored by numerous researchers worldwide [9]. However, peptides derived from natural proteins still require further in-depth investigation.

The spore powder of *G. lucidum* is the generative cells of the Reishi mushroom, known for its powerful health benefits and high nutritional value. Besides polysaccharides, triterpenes and alkaloids, the *G. lucidum* spore protein (GLSP) has attracted great concern due to its remarkable antioxidant capacity [10]. It has been developed as a healthy food additive and pharmaceutical resource due to its functional features. Moreover, the downstream peptides exhibit high biological activity, particularly in inhibiting ACE and DPP-IV, as well as exerting antioxidant effects [11]. Nevertheless, this potential remains largely underutilized and requires further development. Despite the abundance of dietary proteins in GLSP powder, which can normally yield diverse bioactive enzymatic products through enzymatic hydrolysis, isolation, and purification, obtaining specific bioactive peptides from complex protein mixtures remains a time-consuming and costly endeavor. The advancement of bioinformatics has propelled the development of virtual screening techniques based on protein sequences [12]. Many researchers have attempted to employ this strategy for virtual enzymatic hydrolysis to screen peptide sequences and proteases, subsequently identifying superior proteases or discovering novel bioactive compounds by analyzing the resulting peptide fragments [13], thus effectively circumventing the time and material consumption associated with traditional experimental approaches. However, current research predominantly focuses on the potential function prediction of food protein hydrolysates by mixed enzymatic hydrolysis; limited studies have addressed specific bioactive targets under directional enzyme digestion.

In this study, bioinformatics tools, including virtual enzymatic hydrolysis, fragmentomics, and molecular docking, were comprehensively employed to systematically identify antioxidant functional protein hydrolysates from GLSP under effective digestion by target proteases. To achieve that, multi-criteria evaluation methods were adopted in the screening process, including some key parameters surveillance such as peptide and amino acid ratios, potential activity frequency, physicochemical properties, ADMET analysis, as well as antioxidant mechanism. We believe that our findings would be expected to provide both theoretical and practical support for their application in personalized nutrition and modern medical practice.

## 2. Results and Discussion

### 2.1. Potential Biological Activities of the Proteins

**Figure 1:**
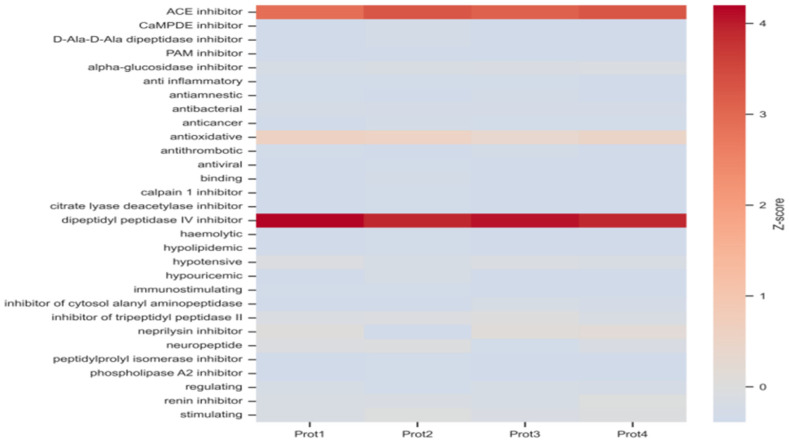
Heat map of predicted bioactivity profiles for four selected proteins from *G. lucidum* spores. The color intensity represents the frequency of bioactive peptide segments (A-value), with higher intensity indicating greater potential for activities such as antioxidant, ACE inhibition, and DPP-IV inhibition.

A group of protein sequences for GLSP was retrieved from the UniProt database and listed in Table S1, which were subjected to predicting their bioactive potential using the UWM module on the BIOPEP-UWM database. After in silico analysis, 30 possible bioactive fragments were swiftly identified across the four selected protein sources (Figure 1). Normally, higher color intensity correlates with increased peptide frequency and elevated Z-score values. It was notable that the aggregate bioactivity frequency was found in Figure 1 within all four protein chains, ranging from 1.50 to 1.80. The detailed data were presented in Table 1. Among them, five bioactivities were highlighted with high occurrence frequencies, including ACE inhibition, DPP-IV inhibition, antioxidant activity, D-Ala-D-Ala dipeptidase inhibition, and hypotensive effects. The most intense color signals were observed for ACE and DPP-IV inhibitory activities on the bioactivity analysis of those protein sequence resources. Specifically, the third-ranked antioxidant activity exhibited a satisfactory Z-score of 1.65, which suggested that the selected proteins derived from GLSP contained potential antioxidant peptides. Consequently, these proteins warrant further investigation via enzymatic hydrolysis to isolate potent antioxidant peptides.

### 2.2. Activity Analysis of Hydrolysis Degree of Proteinase Hydrolysates of GLSP Powder

**Figure 2:**
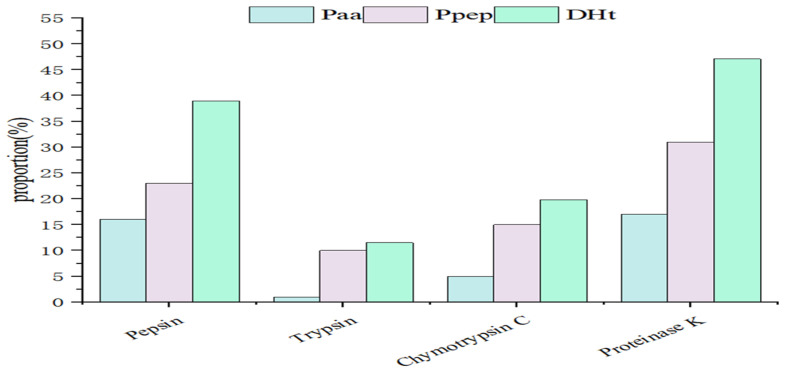
Free amino acid content, peptide content, and theoretical degree of hydrolysis (DHt) of GLSP treated with four enzymes (pepsin, trypsin, chymotrypsin C, and proteinase K).

Discrepancies in cleavage sites allow diverse enzymes to act upon identical protein substrates. This process generates peptide fragments with unique sequences and varied biological activities [14]. Consequently, this study evaluated four specific enzymes: pepsin, trypsin, chymotrypsin, and Proteinase K. We analyzed the degree of hydrolysis (DH_t_) for the resulting fragments. Figure 2 illustrates the distribution profiles of hydrolyzed properties of GLSP after four simulated human gastrointestinal digestive enzymes hydrolysis by using pepsin, trypsin (Tryp), chymotrypsin C (CTRC), and proteinase K (PK). The key parameters were employed to investigate the hydrolysis degree, which were the relative percentages of released free amino acids (P_aa_, light blue bars), polypeptides (P_pep_, light purple bars), and the corresponding calculated theoretical degree of hydrolysis (DH_t_, light green bars) [15].

Generally, the higher DH_t_ ratio it has, the greater enrichment of P_pep_ fragments relative to P_aa_ present in the system, reflecting superior protein degradation and conversion efficiency during the process. There was a remarkable difference in P_aa_ and P_pep_ production among different proteases, attributing to their divergent substrate recognition properties and cleavage site specificities. The PK treatment group exhibited the highest P_pep_ proportion and a suitable P_aa_ level, resulting in the largest DH_t_ ratio among the four groups. This demonstrated that PK could efficiently promote protein degradation and polypeptide accumulation while avoiding excessive hydrolysis into free amino acids with ideal performance. In the pepsin treatment group, the second-highest DH_t_ ratio, with strong protein degradation and polypeptide generation capacity, albeit slightly lower than that of PK. The CTRC group presented a moderate DH_t_ value, with relatively mild polypeptide enrichment ability. And the Tryp group displayed a lower P_pep_ proportion and P_aa_ content, leading to a significantly lower DH_t_ ratio compared with the other groups. This showcase implied its degradation mode led to excessive hydrolysis, which is unfavorable for the relative enrichment of polypeptide fragments. Consequently, PK could maximize DH_t_ and exhibited the best enzymatic hydrolysis efficiency, followed by pepsin, CTRC, and trypsin in a descending order. In order to obtain specific antioxidant derivatives, a combination of PK and pepsin was recommended for the next in silico hydrolysis.

### 2.3. In Silico Enzymatic Digestion of GLSP

Based on the aforementioned analysis, pepsin and proteinase K were identified as the most effective enzymes for digesting the protein chains derived from *G. lucidum* spores. Consequently, these enzymes were selected for in silico digestion using the online virtual enzyme cutting tool—Expasy PeptideCutter (https://web.expasy.org/peptide_cutter/, accessed on 4 December 2025). This approach yielded a total of 1171 peptides generated from the four protein chains.

### 2.4. Amino Acid Analysis of the Hydrolyzed Fragments

The antioxidant potential of the identified peptides is intrinsically linked to their specific amino acid compositions and sequences [12]. Consequently, a systematic analysis of the fragmented components was conducted. Table 2 presents the concentrations of free amino acids within the protein hydrolysates. From that, it was notable that GLSP itself contains high concentrations of hydrophobic amino acids. Specifically, the levels of Ala (9.50%), Leu (8.90%), Val (6.30%), and Gly (6.50%) were prominent, which are fundamental groups to the contribution of antioxidant activity of the peptides [12]. They could dramatically enhance peptide solubility within lipid environments by facilitating proximity to hydrophobic free radicals at the lipid-bilayer interface [16]. Furthermore, the minimal steric hindrance of Gly imparts structural flexibility to the peptide chain. This flexibility allows the molecule to adopt optimal conformations for radical scavenging [17].

Moreover, Asp (6.50%) significantly enhances the metal-chelating capacity of the hydrolysates. The carboxyl groups in Asp side chains act as effective ligands. These ligands sequester transition metal ions, such as Fe^2+^, thereby inhibiting hydroxyl radical formation via the Fenton reaction [18]. Another vital proton or electron donor was also found in the hydrolysates, which were some basic amino acids (Arg (5.40%) and Lys(5.40%)) and hydroxylated residues (Ser(6.30%) and Thr(5.10%)). These polar residues could stabilize reactive oxygen species (ROS) by neutralizing unpaired electrons [19]. Our results reflected that the synergy between hydrophobic “anchor” residues and chemically active “scavenger” residues determines overall efficacy [20]. Such a balanced distribution suggested that two target enzymes could specifically release the antioxidant fragments from GLSP.

### 2.5. In Silico Screening of Antioxidant Peptides

#### 2.5.1. The Relationship Between Property and Key Amino Acids

A total of 218 potentially bioactive sequences after the employment of Peptide Ranker are shown in Table S2, ranging from dipeptides to hexapeptides with scores exceeding 0.5.

It was obvious that the resulting hydrolysates primarily consisted of di- and tri-peptides with 200 to 600 Da Mw. This distribution suggested that these low Mw peptides might possess significant in vivo bioactivity due to direct absorption across the intestinal wall [21]. It is commonly believed that terminal amino acid composition may significantly influence the distribution of isoelectric points (pI), by which higher acidic amino acid contents tend to dissociate hydrogen ions in aqueous solution. Figure S1 presents the pI values of these acidic fragments, which range from 3 to 11. It can be observed that most clusters were within the 3–4 and 5–6 pI ranges, supporting their potential peroxidase-like activity [22]. Another indicator (net charge) was used to unveil its intrinsic antioxidant activity. As shown in Figure S2, the proportion of charges in the 0–2 range was notably higher than that in the −2–0 range. Such a high net positive charge is commonly associated with strong hydrophilicity and favorable solubility, strengthening their antioxidant activity. The reason might be explained by the fact that the positive charge donors are apt to bind to negative free radicals and facilitate the opportunity of metal ion chelation during the scavenging behavior [23].

#### 2.5.2. Physicochemical Feature Prediction

As discussed above, it was established that water solubility directly determines whether a molecule can remain stable in the physiological environment (aqueous phase), be delivered, and interact with its target. Molecules with poor solubility possess virtually no bioavailability. The subsequent Innovagen tool prediction on the obtained peptides led to the screening of 103 peptides with good water solubility. Then, a series of bioinformatic analyses of peptide fragments was conducted to reveal favorable physicochemical attributes, and the results are shown in Table S3. The results indicated that all identified peptide fragments were non-toxic. Three top candidate peptides were excavated to have satisfactory biological stability based on the SVM model analysis, which were PGGKL (0.93), NDSF (0.86), and RRL (0.84). Considering the range of 231.27 Da (GR) to 751.88 Da (DNKCSW), our data elaborated bioactive peptides with lower Mw may enhance the potential bioavailability due to their strong penetration of biological barriers.

#### 2.5.3. Safety Evaluation and Metabolism

ADMET analysis serves as a critical gatekeeping mechanism during early-stage drug discovery. This process systematically evaluates absorption, distribution, metabolism, excretion, and toxicity profiles of double-enzyme hydrolysate. Small-molecule drugs typically enter systemic circulation via oral administration and intestinal absorption. Intestinal permeability was assessed using Caco-2 cell transport and Human Intestinal Absorption (HIA) standards [24]. The blood–brain barrier (BBB) functions as a highly selective endothelial interface. This barrier strictly regulates the influx of substances into neural tissues [21]. Additionally, ideal drug candidates must exhibit high metabolic stability. They should resist rapid degradation by cytochrome P450 (CYP450) enzymes. Furthermore, candidates should not potently inhibit or induce these enzymatic activities. Such stability prevents adverse drug–drug interactions and ensures proper clearance [24].

Table 3 depicts 15 peptides that were further selected for good intestinal permeability. Specifically, YWGK, RY, SDW, EW, NDSF, and KAF achieved high Caco-2 scores. These values were 0.8818, 0.8827, 0.8544, 0.8521, 0.8477, and 0.8181, respectively. Such results suggested that those candidates might possess prominent efficient transportation ability by triggering paracellular or carrier-mediated pathways. The potential of oxidative stress mitigation for the central nervous system was also evaluated. In addition, the potential of these peptides to alleviate oxidative stress in the central nervous system was evaluated. The blood–brain barrier (BBB) scores of RF, KF, RW, KAF, and NDSF ranged from 0.2 to 0.4, highlighting their potential as antioxidant agents. Most peptides exhibited moderate-to-high probability scores for CYP inhibition, suggesting that these peptides are not readily recognized or metabolized by CYP450 enzymes. Consequently, they may resist metabolic degradation in vivo, leading to a prolonged half-life and improved bioavailability. After all the ADMET analyses, seven active peptides remained, including KF, TRF, RMA, LR, KAF, EW and NDSF.

### 2.6. Molecular Docking Scores

The typical Keap1-Nrf2 signaling pathway was adopted to investigate the antioxidant mechanism of the selected peptides with the assistance of molecular docking analysis [12]. In this study, 7 candidate peptides with favorable intestinal absorption were docked against the Keap1 receptor. Table 4 shows that KAF and NDSF exhibited the lowest -CDOCKER ENERGY values among the above seven fragments with −66.5924 and −85.6073 kcal/mol binding energies, respectively. It is generally believed that Arg415, Arg 483, Tyr334, Ser363, Arg380, Asn382, Gln530 and Ser555 were considered to be the active pocket of Keap1 receptor [12,14].

**Figure 3:**
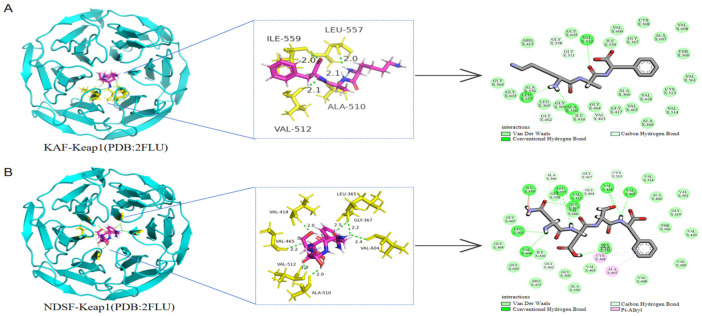
The 3D and 2D molecular docking map of KAF Keap1 receptor with KAF (**A**) and NDSF (**B**).

As expected, the amino acid residues both in KAF and NDSF formed three and twelve conventional hydrogen bonds in the Keap1 binding pocket, respectively, as shown in Figure 3. Notably, the amino group of lysine in KAF and that of aspartic acid in NDSF both could form hydrogen bonds with Leu557 and Ala510 within the Keap1 hydrophobic pocket. Additionally, the carbonyl group of alanine in KAF and the amino group of serine in NDSF concurrently engaged in hydrogen bonding with Val512 of Keap1. Hydrogen bonds serve as the predominant polar interaction between Keap1 and peptide ligands [25], suggesting that both KAF and NDSF possess considerable antioxidant potential. Furthermore, the phenyl ring of phenylalanine within NDSF was uniquely observed to form additional alkyl interactions with Cys368 and Ala607. Concurrently, the carbonyl group of aspartic acid was found to interact with Arg415, contributing to the overall binding with Keap1. Apart from that, cysteine residues such as Cys368, along with arginine residues, were also critically involved in inhibiting Nrf2 degradation [26]. Both KAF and NDSF can form hydrogen bonds and hydrophobic interactions with key residues within the Keap1 active pocket, including Leu557, Ala510, Val512, Arg415, and Cys368, suggesting that they may interfere with the residues involved in Keap1’s recognition of Nrf2.

The ETGE motif (Glu-Thr-Gly-Glu) forms a strong electrostatic/hydrogen-bond network with Arg415 and Arg483 of Keap1 through its acidic side chains, which is crucial for maintaining the high-affinity anchoring of Nrf2. Our docking results clearly show that the side-chain oxygen atoms of Asp (aspartic acid) and Ser (serine) in NDSF form hydrogen bonds precisely with Arg415. Thus, NDSF may competitively occupy the canonical binding cavity of the ETGE motif, thereby weakening the Keap1-mediated anchoring of Nrf2 and promoting the release of Nrf2 from the complex. Xu et al. [27] demonstrated that gingerol and shogaol can spontaneously interact with the active site of the Keap1 protein, which consists of specific residues including Arg415, Phe478, Arg483, Phe577, Tyr572, Tyr334, and Tyr525. The binding sites identified in the present study are consistent with these residues, thereby further validating the reliability of this binding mode.

On the other hand, the DLG motif (Asp-Leu-Gly) exhibits lower affinity, and its binding relies on hydrophobic residues such as Leu557, Ala510, and Val512, as well as the hydrogen bond contributed by Tyr334. The two core peptides identified in our study are both able to form hydrogen bonds or hydrophobic contacts with Leu557, Ala510, and Val512. This competitive binding might specifically block the low-affinity interaction of the DLG motif, potentially mimicking the “latch-opening” effect induced by Cys273/Cys288 modification under oxidative stress, and could thereby inactivate the ubiquitination of Nrf2 without completely disrupting ETGE binding. These results suggest that both peptides may possess potential antioxidant activity, and that the tetrapeptide (NDSF), by establishing a robust multi-dimensional interaction network, might be more effective than KAF in alleviating oxidative stress.

### 2.7. Evaluation of Free Radical Scavenging Capacity of Two Antioxidant Peptides

**Figure 4:**
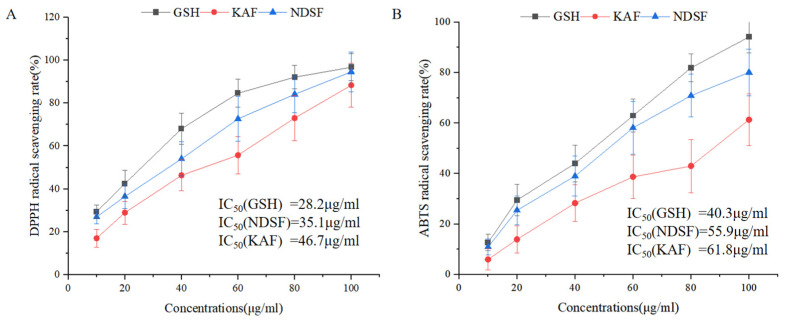
In vitro antioxidant activities of KAF and NDSF. (**A**) DPPH radical scavenging activities and (**B**) ABTS radical scavenging activities.

The DPPH and ABTS assays are well-established methods for evaluating the antioxidant capacity of bioactive peptides in vitro. In this study, the radical scavenging activities of NDSF, KAF, and GSH against ABTS and DPPH were quantitatively assessed over a concentration range of 0–100 μg/mL in Figure 4. All samples exhibited concentration-dependent DPPH scavenging capacity. At 60 μg/mL, NDSF showed stronger radical activity (72.6%) than KAF (55.7%), although that value was lower than GSH (84.6%). The IC_50_ values for NDSF and KAF were 35.1 μg/mL and 46.7 μg/mL, compared to GSH (28.2 μg/mL). Similar trends emerged in the ABTS assay with the increase in these two peptide concentrations. The scavenging capacity of NDSF could acquire 58.1% when the concentration was increased to 60 μg/mL, distinct from 38.7% scavenging rate for KAF. And the IC_50_ values for NDSF and KAF were 55.9 μg/mL and 91.8 μg/mL.

Owing to the existence of hydrophobic (Phe) and acidic (Asp) amino acids in the tetrapeptide, this feature would help the tetrapeptide penetrate lipid bilayers to attack harmful free radicals and promote the oxidative activity of peroxidase. In addition, the high net positive charges inside might be beneficial for the electron donation during the scavenging process. Moreover, due to the Ser residue being adjacent to Phe, the carbonyl group can easily form super conjugation system with phenyl ring. That unique structure would accelerate the hydrogen bond formation when NDSF encounters the bioactive pockets of the Keap1 receptor. In which the peptides they screened also had higher contents of hydrophobic and acidic amino acids, and showed similar results. Consequently, this selected tetrapeptide (NDSF) had a stronger scavenging capacity than thKAF on both DPPH and ABTS.

### 2.8. Quantum Chemical Analysis

#### 2.8.1. Frontier Molecular Orbitals

Frontier molecular orbitals (FMOs) are widely utilized in quantum chemical calculations to characterize antioxidant activity. Specifically, the highest occupied molecular orbital (HOMO) and lowest unoccupied molecular orbital (LUMO) serve as essential parameters. These metrics evaluate antioxidant potential and identify active sites within peptides. The HOMO predicts regions with robust electron-donating capacity. High electron cloud density within the HOMO highlights groups with a strong tendency to donate electrons. Consequently, sites with potent free radical scavenging ability are directly identifiable through these locations [28].

**Figure 5:**
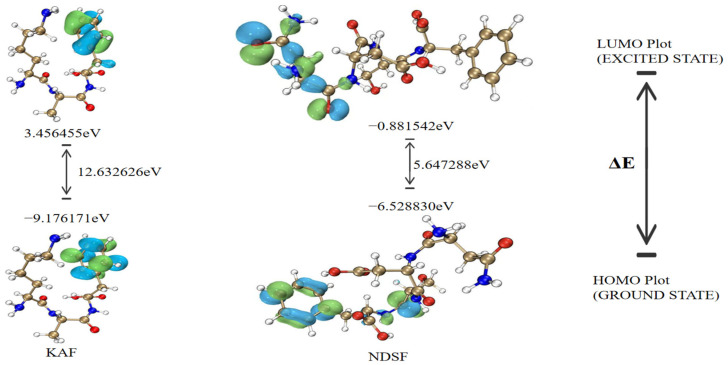
The HOMO and LUMO distributions of KAF and NDSF. Note: The green and blue regions in the amino acid sequence indicate those that contribute to HOMO. ΔE refers to the energy gap from HOMO to LUMO.

Figure 5 illustrates the energy levels of HOMO, HOMO-1, LUMO, and LUMO-1 for KAF and NDSF. The HOMO of KAF is primarily localized on Phe, while that of NDSF is concentrated on Ser. These findings suggested that these specific residues are prone to electron loss during radical interactions. The energy gap (ΔE) between HOMO and LUMO reflects molecular bioactivity. The result showed the tetrapeptide NDSF exhibited an ΔE of 5.647288 eV, whereas the tripeptide KAF showed an ΔE of 12.632626 eV. The narrower ΔE in NDSF proved its enhanced chemical reactivity and structural flexibility [29]. Therefore, NDSF possesses superior theoretical antioxidant capacity compared to KAF. Interestingly, both HOMO and LUMO in KAF were contributed by the Phe residue. The reason may be explained by the fact that the phenyl side chain of Phe readily forms an independent pi-conjugated system with a low energy gap, while the positively charged Lys and saturated Ala lack competing low-energy orbitals [30]. This classic orbital localization phenomenon is controlled by the inherent chemical properties of amino acids [31].

#### 2.8.2. Active Sites of Antioxidant Peptides

Charge distribution fundamentally determines the reactive sites of antioxidant peptides. These active regions are characterized by concentrated HOMO electron density and highly electronegative electrostatic potential (ESP) [32]. Such parameters evaluate the capacity of peptides to donate electrons or hydrogen atoms during radical scavenging.

In NDSF, the active sites were identified as C17 and N25 on the serine amino side chain, along with all carbon atoms on the phenylalanine phenyl ring. For KAF, by contrast, the active sites comprised the phenyl ring carbons and the carbonyl oxygen. According to Wang et al. (2025), the negatively charged nitrogen on the serine amino side chain serves as a key hydrogen-donating site [32,33], implying that nitrogen and carbon atoms within the HOMO region contribute to peptide antioxidant activity. To further characterize intramolecular active sites, molecular electrostatic potential (ESP) and average local ionization energy (ALIE) were employed, with ESP serving as a fundamental descriptor of molecular reactivity and interaction potential [34,35].

**Figure 6:**
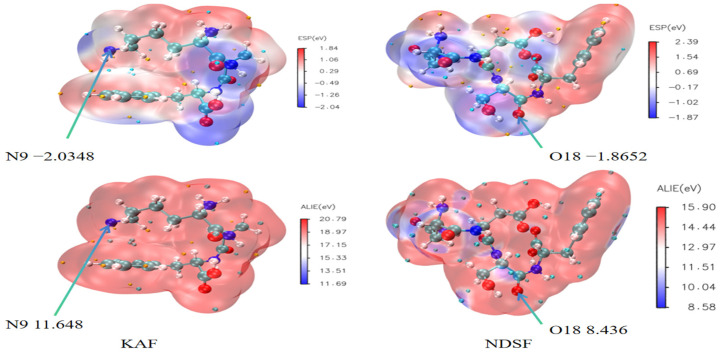
Isosurface profiles of electrostatic potential (ESP) and average ionization energy (ALIE) at electron densities of 0.01 eV. Note: blue spheres represent local minima, while orange-red spheres mean local maxima.

On the ESP surface, red regions represent positive values conducive to nucleophilic attack. Conversely, blue regions denote negative values favoring electrophilic attack and proton acceptance [36,37]. ALIE functions as an auxiliary tool to refine these predictions. Sites featuring the most negative ESP and the lowest ALIE are highly susceptible to free radical scavenging. A highly negative ESP signifies maximum electron density, acting as a preferred site for attack by electron-deficient radicals. Meanwhile, the lowest ALIE indicates minimal energy required for electron loss. When ESP and ALIE results converge, the site is identified as a hotspot for radical scavenging. In NDSF, O18 on the serine side-chain hydroxyl exhibited the strongest electronegativity and lowest ALIE (Figure 6). For KAF, these properties were localized at N9 on the lysine terminal amino group. This analysis further confirmed the active sites of N25-Ser in NDSF and N9-Lys in KAF, displaying pronounced nucleophilic activity via single-electron transfer. In addition, O18 on the Asp carboxyl group and C17/N25 on the Ser amino side chain in NDSF could serve as primary electron-donating centers as well. This aligns with the results obtained from HOMO analysis [38]. Based on these criteria, NDSF demonstrates greater antioxidant activity than KAF. This conclusion is entirely consistent with the HOMO analysis findings.

### 2.9. Cytotoxicity of the Core Peptides in HepG2 Cells

HepG2 cells exhibit metabolic and biotransformation activities similar to those of human hepatocytes and are therefore widely used to evaluate drug metabolism and cellular uptake processes. Although molecular docking is valuable for initial screening, it may not fully capture the complexity of interactions in a physiological environment. To validate the potential of the screened peptides to inhibit the Keap1-Nrf2 interaction under physiological conditions, we performed an H_2_O_2_-induced oxidative stress assay to further assess the actual effects of KAF and NDSF on oxidative stress and cellular damage in the HepG2 cell model. Synthetic peptides, which showed better performance in both in vitro and digestion models, were selected for establishing the cell model.

**Figure 7:**
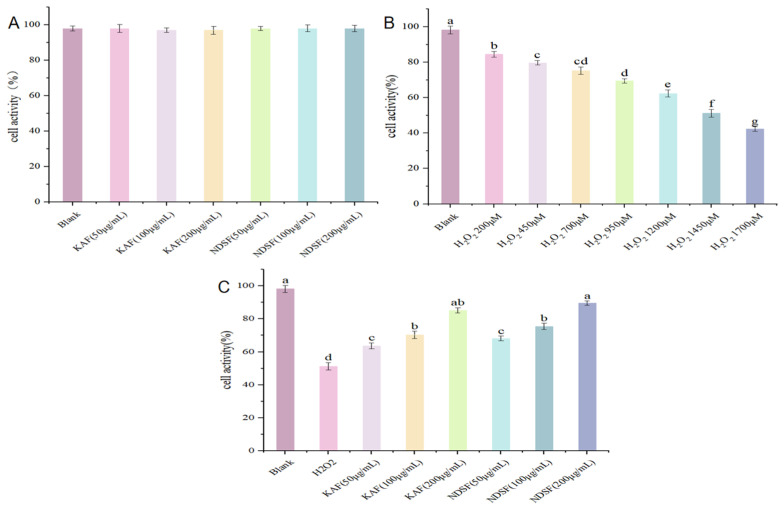
Cytotoxicity and cytoprotective effects of KAF and NDSF on cells. (**A**) Cell viability after treatment with different concentrations of KAF and NDSF alone. (**B**) Cell viability under different concentrations of H_2_O_2_ stimulation. (**C**) Protective effects of KAF and NDSF on H_2_O_2_-induced cell viability reduction. Values are expressed as mean ± SD (*n* = 3). Bars with different lowercase letters are significantly different (*p* < 0.05) according to one-way ANOVA followed by Tukey’s multiple range test.

Before the main experiment, HepG2 cells were treated with different concentrations of the peptides (50–200 μg/mL) for 24 h. The cytotoxicity results, shown in Figure 7A, indicated that the proliferation rates of the peptide-treated groups were not adversely affected compared with the control group, suggesting that neither KAF nor NDSF exhibited noticeable toxic effects. Therefore, these concentrations of the antioxidant peptides were used for subsequent experiments. Previous studies have shown that short peptides with low molecular weight and relatively high hydrophobicity are more likely to dissolve in and penetrate the lipid bilayer of cell membranes, thereby enhancing their antioxidant activity [39]. Of note, it has also been reported that KF10, at concentrations ranging from 50 to 200 μg/mL, similarly showed no toxic effects and even promoted the proliferation of HepG2 cells [40].

### 2.10. Establishment of the H_2_O_2_-Induced Oxidative Damage Model in HepG2 Cells

In establishing an oxidative stress model, the optimal cell viability is typically around 50–60%. Too low a concentration of H_2_O_2_ fails to induce significant oxidative damage, whereas too high a concentration causes irreversible cell injury; neither extreme is suitable for establishing a reliable oxidative stress model [41]. To investigate the reparative effects of the peptides on damaged cells, we first treated the cells with H_2_O_2_ at concentrations ranging from 200 to 1700 μmol/L for modeling (Figure 7B). Treatment with 1450 μmol/L H_2_O_2_ for 4 h significantly reduced cell viability to 51.2 ± 2.23%, a level that meets the requirements for inducing oxidative damage while preserving sufficient cell viability for subsequent experiments. In addition, H_2_O_2_-treated cells exhibited mild shrinkage and detachment, along with a decrease in cell density.

### 2.11. Protective Effect of the Core Peptides Against H_2_O_2_-Induced Oxidative Damage in HepG2 Cells

The reparative effects of the peptides are presented in Figure 7C. Compared with the model group treated with 1450 μM H_2_O_2_, cells pretreated with the peptides showed less damage. After 24 h of pre-incubation, the protective effect was most pronounced at a concentration of 200 μg/mL, where cell viability reached 89.6 ± 1.27%. This represents a significant increase of 38.4 ± 1.92% compared with the H_2_O_2_-only group (51.2 ± 2.23%).

### 2.12. Effects of the Core Peptides on Antioxidant Enzymes in H_2_O_2_-Induced HepG2 Cells

**Figure 8:**
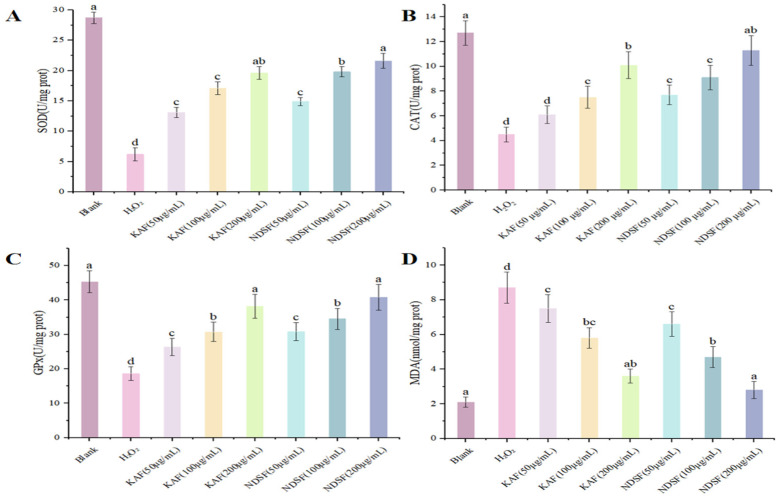
Antioxidant effects of KAF and NDSF in H_2_O_2_-stimulated oxidative damage in cells. (**A**) SOD activity, (**B**) CAT activity, and (**C**) GPx activity were measured to evaluate the function of the endogenous antioxidant enzyme system. (**D**) MDA content was determined as a marker of lipid peroxidation and oxidative membrane damage. Values are expressed as mean ± SD (*n* = 3). Bars with different lowercase letters are significantly different (*p* < 0.05) according to one-way ANOVA followed by Tukey’s multiple range test.

H_2_O_2_ induces the activation of antioxidant enzymes within the cellular oxidative/non-oxidative systems in HepG2 cells to protect against oxidative damage. These antioxidant enzymes include GPx, CAT, SOD, and MDA. As shown in Figure 8A–C, the levels of SOD, CAT, and GSH-Px were significantly inhibited in the H_2_O_2_ group. This decrease can be attributed to the activation of the antioxidant defense system in HepG2 cells stimulated by H_2_O_2_, leading to a higher demand for these enzymes. In the peptide-treated groups, the activities of CAT, SOD, and GPx showed a decreasing trend with increasing peptide concentration, which is likely due to the potent free-radical-scavenging capacity of the synthetic antioxidant peptides KAF and NDSF.

Compared with the blank control group, the H_2_O_2_ model group exhibited significantly decreased activities of SOD, CAT, and GPx (from 28.7, 12.7, and 45.3 U/mg prot to 6.2, 4.5, and 18.6 U/mg prot, respectively). Pretreatment with KAF or NDSF for 12 h reversed these changes in a concentration-dependent manner. At 50 and 100 μg/mL, both peptides markedly increased SOD, CAT, and GPx activities while reducing MDA content, as shown in Figure 8D; NDSF was slightly more effective than KAF. At the highest concentration tested (200 μg/mL), KAF restored CAT and GPx to levels comparable to those of the blank control group (11.2 and 38.2 U/mg prot, respectively). However, SOD activity (19.6 U/mg prot) remained significantly lower than that of the blank control (28.7 U/mg prot), and MDA content (3.6 U/mg prot) did not return completely to the normal range. In contrast, under the same concentration, NDSF restored SOD, CAT, and GPx to 21.6, 12.0, and 40.8 U/mg prot, respectively, and reduced MDA to 2.8 U/mg prot. These results indicate that both KAF and NDSF effectively enhance the endogenous antioxidant defense system and inhibit lipid peroxidation, with NDSF exhibiting a more comprehensive protective effect.

## 3. Materials and Methods

### 3.1. Materials and Chemicals

2,2′-Azino-bis (3-ethyl benzothiazoline-6-sulfonic acid) diammonium salt (ABTS, 98%), 1,1-diphenyl-2-picrylhydrazyl (DPPH, 98%), Potassium persulfate and glutathione (GSH) were purchased from Shanghai Aladdin Biochemical Technology Co., Ltd. (Shanghai, China). Cell line: HepG2 cells (human hepatocellular carcinoma cells) were obtained from Wuhan Pricella Life Science & Technology Co., Ltd. (Wuhan, China). Main reagents: DMEM high-glucose medium, fetal bovine serum (FBS), penicillin-streptomycin, 0.25% trypsin-EDTA, phosphate-buffered saline (PBS), Cell Counting Kit-8, H_2_O_2_, DCFH-DA probe, and assay kits for SOD, MDA, CAT, and GSH-Px (From Beyotime Biotechnology, Shanghai, China). Instruments: CO_2_ incubator, microplate reader (450 nm and fluorescence at 488/525 nm), inverted fluorescence microscope, and low-temperature centrifuge (Thermo Fisher Scientific Inc., Waltham, MA, USA). All other reagents and chemicals were analytical grade.

### 3.2. Potential Biological Activity

Basic proteins of GLSP were selected from the dataset in the UniProt database (https://www.uniprot.org/). The potential biological activities of these four confirmed protein chains were subsequently predicted using the “potential biological activity spectrum” tool in the BIOPEP-UWM database. The relative bioactivities of peptides can be predicted, including antioxidant, angiotensin-converting enzyme (ACE) inhibitory, and dipeptidyl peptidase-IV (DPP-IV) inhibitory activities, which occurred with the highest frequency. According to Formula (1), the evaluation parameter (A) was calculated, which is defined as the frequency of occurrence of peptides with a specific activity within the protein sequence.

A = a/N(1)

where a means the number of peptides with a given activity in the protein sequence; N represents the number of amino acid residues in the protein.

### 3.3. Virtual Enzymatic Hydrolysis of GLSP

In silico enzymatic hydrolysis of the four selected GLSP was performed using the online virtual cleavage tool Expasy PeptideCutter (https://web.expasy.org/peptide_cutter/, accessed on 5 December 2025), and the resulting peptides were subjected to amino acid composition analysis via the Expasy ProtParam tool (https://web.expasy.org/protparam/, accessed on 5 December 2025). Concurrently, the theoretical degree of hydrolysis (DH_t_), the theoretical free amino acid ratio (P_aa_), and the theoretical peptide ratio derived from the cleavage fragments (P_pep_) were calculated according to Equations (2)–(4) to evaluate the extent of enzymatic hydrolysis, the degree of amino acid liberation, and the proportion of potential bioactive peptides derived from GLSP proteins.

DH_t_(%) = n/N × 100 (2)

P_aa_(%) = n_aa_/N × 100 (3)

P_pep_(%) = DH_t_ − P_aa_ (4)

where N, n and n_aa_ correspond to total amino acids in the protein sequence, total hydrolyzed protein fragments and total free amino acids, respectively.

### 3.4. Enzymatic Fragment Activity Analysis

The proteolytic peptides from GLSP powder were analyzed using the PeptiderRanker server (http://distilldeep.ucd.ie/PeptideRanker/, accessed on 5 December 2025), where their bioactivity scores were evaluated to identify potential bioactive peptides ranging from dipeptides to hexapeptides, with free amino acids and polypeptides specifically excluded from the screening. The scores range from 0 to 1, with a threshold of 0.5 (i.e., peptides with a score >0.5). A score close to 1 indicates a higher likelihood that the peptide has bioactive properties, including antioxidant properties [42].

### 3.5. Online Screening of Enzymatic Hydrolysates

The amino acid sequences with a bioactivity score greater than 0.5 obtained from the above step were subsequently subjected to prediction of solubility, allergenicity, and toxicity using Innovagen (http://www.innovagen.com/proteomics-tools, accessed on 5 December 2025), Allertop (https://www.ddg-pharmfac.net/AllerTOP/, accessed on 5 December 2025), and Toxinpred (https://webs.iiitd.edu.in/raghava/toxinpred/, accessed on 5 December 2025), respectively. Peptide sequences predicted to be non-toxic, non-allergenic, and with favorable solubility were selected for further analysis [43]. Subsequently, their molecular weight, net charge, and isoelectric point were predicted using Pepdraw (https://www.pepdraw.com/) and the Expasy Compute pI/Mw tool (https://web.expasy.org/compute_pi/, accessed on 5 December 2025).

### 3.6. Human Absorbability of Enzymatic Hydrolysates

The amino acid sequences of the enzymatically hydrolyzed fragments derived from the screened peptides—which exhibited favorable water solubility, non-toxicity, and non-allergenicity—were converted into SMILES notations and subsequently imported into AdmetLab 2.0 (https://admet.scbdd.com/) for standard analysis [44]. The absorbability of these GLSP hydrolysate fragments (excluding free amino acids) was evaluated by assessing their human intestinal absorption (HIA), blood–brain barrier (BBB) penetration, and cytochrome P450 (CYP2C9, CYP2D6, CYP3A4) metabolism. The HIA parameter distinguishes between good intestinal absorption (HIA+) and poor intestinal absorption (HIA−).

### 3.7. Molecular Docking

The 2D structure of the polypeptide was constructed using ChemBioDraw 14.0 and then subjected to get its optimal 3D conformation via ChemBio Ultra 14.0 after following subsequent energy minimization treatment.

The natural crystal structure of Keap1 (PDB ID: 2FLU) was retrieved from the protein data bank. We used Pymol2.6 to remove water molecules, hydrogen atoms, and original ligands from the receptor. Molecular docking was then conducted using Autodock Vina 13.1. The conformation with the lowest binding energy was selected for model development. Finally, we utilized Discovery Studio 2020 for visualization. This evaluated the antioxidant activity and binding modes of the GLSP.

### 3.8. Synthesis of Antioxidant Peptides

The identified antioxidant peptides derived from GLSP were synthesized by Shenggong Bioengineering (Shanghai, China) Co., Ltd., with a purity of at least 97%.

### 3.9. Determination of Antioxidant Capacity

#### 3.9.1. DPPH Assay

The DPPH radical scavenging capacity was carried out according to the reference method with slight modifications [45]. A DPPH ethanol solution with a concentration of 2 mmol/L was prepared and stored in the dark. A volume of 100 µL of each synthesized peptide concentration or GSH solution was dispensed into a 96-well plate and mixed with 100 µL of DPPH stock solution. The reaction was carried out in the dark at room temperature for 30 min. The absorbance was measured at 517 nm. Glutathione (GSH) was used as the positive control. The formula for the DPPH radical scavenging capacity is shown in Equation (5):

DPPH radical scavenging capacity/% = [1 − (A_1_ − A_2_)/A_3_] × 100% (5)

where A_1_ represents the absorbance of the sample to be tested or the positive control; A_2_ refers to the blank absorbance, which is the reaction of the sample to be tested or the positive control mixed with anhydrous ethanol; and A_3_ means the control absorbance, which is the reaction of deionized water mixed with the DPPH solution.

#### 3.9.2. ABTS Assay

Determination of ABTS cation radical scavenging capacity was conducted following the reference [45]. Briefly, a 5 mmol/L ABTS solution was mixed with 7 mmol/L K_2_S_2_O_8_ solution and kept in the dark at room temperature for 12 h. For the preparation of the free radical reagent, the solution was diluted with deionized water to yield an absorbance of 0.7 ± 0.02. Then, 200 μL of different concentrations of polypeptide solution or GSH solution was added to 96-well plates, followed by the addition of 200 μL of ABTS stock solution. After the dark reaction for 6 min, the absorbance was recorded at 734 nm, and the formula for calculating the scavenging capacity was shown as (6):

ABTS cation radical scavenging capacity/% = [(A_0_ − A_1_)/A_0_] × 100% (6)

In the formula, A_1_ is the absorbance of the sample to be tested or the positive control; A_0_ is the blank absorbance. The sample to be tested, or the positive control, is mixed and reacted with the ABTS solution.

### 3.10. Quantum Chemical Calculations

The electronic donation capacity of two selected peptides was further investigated by DFT computations using Gaussian 16 W after the geometric optimization by Gaussian View 5.0 based on B3LYP/6-31G(d,p), according to the previous methods [46,47]. The highest electron occupied orbit (EHOMO) and the lowest electron unoccupied orbit (ELUMO) were employed to evaluate their electron energy. Electrostatic potential (ESP) and average local ionization energy (ALIE) were adopted to explore antioxidant sites of the target by Multiwfn 3.8 calculation and the isosurface maps were visualized by using visual molecular dynamics (VMD 1.9.3).

### 3.11. Cell Culture

Complete cell culture medium was prepared by adding 90% DMEM, 10% fetal bovine serum (FBS), and 1% penicillin-streptomycin (100×) solution to a culture flask. Cryopreserved cells were quickly thawed in a 37 °C water bath. Once completely thawed, 1 mL of complete medium was added to the cell suspension, and the mixture was centrifuged at 1500 rpm for 5 min. The supernatant was then removed. Next, 4 mL of complete medium was added to a 225 cm^3^ cell culture flask, and the cells were placed in a 37 °C, 5% CO_2_ incubator for adherent culture for 24 h. After thawing, the HepG2 cells were transferred to a 25 cm^3^ culture flask containing 4 mL of complete medium. The flask was kept in a 37 °C, 5% CO_2_ incubator.

### 3.12. Cytotoxicity Assay

HepG2 cells were harvested by trypsinization and seeded into 96-well plates. Each well received 100 μL of cell suspension at a density of 1 × 10^5^ cells/mL, and the plates were incubated for 24 h at 37 °C in a 5% CO_2_ incubator. The biosynthetic peptides were then added to the cells at concentrations of 50, 100, and 200 μg/mL. After 12 h of incubation, cell viability was immediately determined using the CCK-8 assay. Cell viability was calculated using the following formula:

Cell viability (%) = (A_1_ − A_0_)/(A_2_ − A_0_) × 100% (7)

where A_1_ represents the experimental group (cells cultured in medium containing CCK-8 and the test peptide), A_2_ represents the control group (cells cultured in medium containing CCK-8 but no test peptide), and A_0_ represents the blank group (medium containing CCK-8 without cells or test peptide).

### 3.13. Establishment of the Cell Oxidative Damage Model

HepG2 cells were seeded into 96-well plates at a density of 5 × 10^4^ cells per well in 100 μL of culture medium. After 24 h of incubation, the original medium was discarded, and the cells were treated with 100 μL of serum-free DMEM high-glucose medium containing various concentrations of H_2_O_2_ (200, 450, 700, 950, 1200, 1450, and 1700 μmol/L) for 4 h. Cell viability was then measured using the CCK-8 assay. The H_2_O_2_ concentration that reduced cell viability to approximately 50% was selected as the optimal working concentration for inducing the oxidative damage model.

### 3.14. Protective Effect of the Core Peptides Against H_2_O_2_-Induced Oxidative Damage in HepG2 Cells

Cells were treated following the same procedure as described above. In 96-well plates, the following groups were set up: a control group, an H_2_O_2_-only model group, and experimental groups treated with low, medium, and high doses of the antioxidant peptides. Each well received 100 μL of cell suspension. After 24 h of incubation, the original culture medium was discarded, and the cells were gently washed twice with PBS. For the experimental groups, 100 μL of serum-free DMEM high-glucose medium containing different concentrations of the peptides (50, 100, and 200 μg/mL) was added. The control and model groups received the same volume of serum-free DMEM high-glucose medium without peptides. Following another 24 h of incubation, the medium was removed, and the cells were washed twice with PBS. Then, 100 μL of 1450 μmol/L H_2_O_2_ was added to both the experimental and model groups, while the control group received 100 μL of serum-free DMEM high-glucose medium. After 4 h of further incubation, cell viability was assessed using the CCK-8 assay.

### 3.15. Measurement of Intracellular SOD, CAT, GSH-Px, and MDA Levels in HepG2 Cells

HepG2 cells were seeded into 6-well plates at a density of 1 × 10^6^ cells/mL and treated according to the grouping and procedures described in Section 3.14. After washing with PBS, the culture plates were placed on ice, and 100 μL of RIPA lysis buffer was added to each well. The lysates were centrifuged at 11,000 rpm for 15 min at 4 °C, and the supernatants were collected and stored at −80 °C. Protein concentrations were determined using a BCA protein assay kit. The activities of MDA, GSH-Px, SOD, and CAT in the cell lysates were then measured following the instructions provided with the respective assay kits.

### 3.16. Statistical Analysis

All data were expressed as mean ± standard deviation (SD) from at least three independent experiments. Statistical analyses were performed using GraphPad Prism 8.0 software. Differences between groups were assessed by one-way analysis of variance (ANOVA) followed by Tukey’s post hoc test. A value of *p* < 0.05 was considered statistically significant, while *p* < 0.01 was regarded as highly significant.

## 4. Conclusions

This study aimed to explore the hydrolysis products of GLSP proteins with antioxidant properties. Target protein chains were first selected from protein databases, followed by virtual enzymatic hydrolysis [48]. The research revealed that 1171 peptide derivatives from *G. lucidum* spore powder treated with specific pepsin and protease K were determined to contain abundant hydrophobic and acidic amino acids, suggesting their enzymatic hydrolysis products may exhibit broad biological activity [49]. Through bioinformatics assessment, 15 peptide compounds exhibited favorable digestive absorption characteristics without significant toxicity observed. After novelty prediction screening, 7 peptides were identified for further investigation. These results highlighted the convenience and efficiency of virtual screening methods to rapidly get the desired targets. Molecular docking analysis further identified two peptides, KAF and NDSF, as exhibiting the lowest docking energies. Both peptides were capable of binding to the Leu557, Ala510, and Val512 residues within the bioactive pocket of the Keap1 protein via hydrogen bonds, suggesting their potential antioxidant activity. Quantum chemistry analysis revealed that the tetrapeptide NDSF had a lower ΔE of 5.647288 eV, compared to the ΔE of 12.632626 eV in tripeptide KAF [46]. The analysis of ESP and ALIE further identified key antioxidant active sites for both peptides: O18 of Ser in the NDSF chain and N9 of Lys in the KAF chain. An in vitro antioxidant test validated the capacity of the virtual screening strategy, in which both NDSF and KAF exhibited potent DPPH and ABTS radical scavenging rates, and the former manifested even stronger performance. Meanwhile, the significant improvement in antioxidant enzyme activities observed in both the in vitro antioxidant assays and the cellular experiments further supports the validity of our virtual screening strategy. There are several limitations of this study that should be acknowledged. First of all, the *Ganoderma lucidum* spore protein sequences used for in silico enzymatic hydrolysis were retrieved from the UniProt database, which does not yet cover the entire proteome. This incompleteness may have led to the omission of some potentially active peptides. Next, the interactions between NDSF and key Keap1 residues (e.g., Arg415, Cys368) revealed by molecular docking are purely computational predictions. They still await experimental validation using techniques such as site-directed mutagenesis or surface plasmon resonance. The BBB scores (0.2–0.4) from our ADMET predictions only hint at the possibility that these peptides might cross the blood–brain barrier. Any true neuroprotective effect would need to be verified in animal models, such as MPTP-induced Parkinson’s disease mice. Moreover, the changes in SOD, CAT, GPx, and MDA levels observed in the cell experiments hardly reflect the effects of in vivo enzyme induction. Consequently, further animal studies are strongly required to confirm those findings.

## Figures and Tables

**Table 1:** Active frequencies of the protein chains from four types of *G. lucidum* spore powder.

Protein	Activity Type	a	N	A(a/N)
Q92429	Antioxidative	30	200	0.150
	ACE inhibitory	106	200	0.530
	(DPP-IV) inhibitory activity	120	200	0.600
B4YA15	Antioxidative	51	360	0.142
	ACE inhibitory	150	360	0.417
	(DPP-IV) inhibitory activity	180	360	0.500
G9BIY1	Antioxidative	60	400	0.150
	ACE inhibitory	200	400	0.500
	(DPP-IV) inhibitory activity	250	400	0.625
A0SJQ5	Antioxidative	95	467	0.203
	ACE inhibitory	260	467	0.557
	(DPP-IV) inhibitory activity	411	467	0.880

Active frequencies of four protein chains in antioxidant, ACE inhibitory, and DPP-IV inhibitory activity. where a means the number of peptides with a given activity in the protein sequence; N represents the number of amino acid residues in the protein.

**Table 2:** Amino acid composition of proteins from *G. lucidum* spore powder.

Amino Acid Species	Proportion of Total Amino Acids	Amino Acid Species	Proportion of Total Amino Acids
Ala	9.50%	Leu	8.90%
Arg	5.40%	Lys	5.40%
Asn	4.20%	Met	1.90%
Asp	6.50%	Phe	4.70%
Cys	1.60%	Pro	4.10%
Gln	3.90%	Ser	6.30%
Glu	5.30%	Thr	5.10%
Gly	6.50%	Trp	1.70%
His	3.10%	Tyr	3.80%
Ile	5.80%	Val	6.30%

Amino acid composition of the four selected protein chains from *G. lucidum* spore powder. The values are expressed as the percentage of each amino acid relative to the total amino acid content.

**Table 3:** Predicted ADMET properties of the screened peptides: Caco-2 cell permeability, cytochrome P450 (CYP2C9, CYP2D6, CYP3A4) inhibition, human intestinal absorption (HIA), and blood–brain barrier (BBB) permeability.

Peptides	Caco-2 Permeability	CYP2C9	CYP2D6	CYP3A4	HIA	BBB
RF	0.7607^+^	0.7407	0.7407	0.7855	0.7912^+^	0.3368^+^
KF	0.7064^+^	0.8614	0.8056	0.7990	0.8238^+^	0.3011^+^
RW	0.762^+^	0.8206	0.7297	0.7260	0.9644^+^	0.3368^+^
YWGK	0.8818^+^	0.8707	0.7212	0.5725	0.9107^+^	0.5313^+^
RRCW	0.7835^+^	0.8076	0.7455	0.7124	0.8147^+^	0.5161^+^
TRF	0.7835^+^	0.8076	0.7455	0.7124	0.8147^+^	0.5161^+^
SDW	0.8544^+^	0.8678	0.8133	0.7135	0.7926^+^	0.4165^+^
RL	0.752^+^	0.7931	0.7654	0.6825	0.8271^+^	0.5980^+^
RMA	0.7039^+^	0.7961	0.7731	0.6708	0.5799^+^	0.5787^+^
RY	0.8827^+^	0.7932	0.7649	0.6865	0.6163^+^	0.4121^+^
LR	0.752^+^	0.7931	0.7654	0.6825	0.8271^+^	0.5980^+^
YR	0.8827^+^	0.7932	0.7649	0.6865	0.6163^+^	0.6121^+^
KAF	0.8181^+^	0.8162	0.8236	0.7385	0.7042^+^	0.2368^+^
EW	0.8521^+^	0.8725	0.8372	0.6804	0.7414^+^	0.6309^+^
NDSF	0.8477^+^	0.8649	0.8481	0.7807	0.7860^+^	0.2133^+^

ADMET properties of peptides. Caco-2, Caco-2 permeability; CYP2C9/CYP2D6/CYP3A4, cytochrome P450 2C9/2D6/3A4 inhibition; HIA, human intestinal absorption; BBB, blood–brain barrier permeability. The superscript “+” denotes the predicted active property of the corresponding index.

**Table 4:** Molecular docking-derived binding energies (-CDOCKER ENERGY, kcal/mol) of the screened peptides and glutathione (GSH) with the Keap1 receptor protein.

Peptides	-CDOCKER ENERGY (kcal/mol)
KF	57.6540
TRF	64.5823
RMA	62.7315
LR	52.9046
KAF	66.5924
EW	55.9265
NDSF	85.6073
GSH	58.8950

-CDOCKER energies were obtained from molecular docking simulations using the Keap1 structure (PDB: 2FLU). Lower values denote more favorable binding. GSH was included as a positive control. These energies serve for qualitative comparison among the tested peptides.

## Data Availability

Data are contained within the article and Supplementary Materials.

## Supplementary Materials

The following supporting information can be downloaded at https://www.mdpi.com/article/10.3390/molecules31122157/s1, Figure S1: Isoelectric point distribution of peptides; Figure S2: The Net charge of the peptide; Table S1: The basic information of four selected protein chains derived from the spore powder of Ganoderma lucidum title; Table S2: The peptide activity produced by the enzymatic digestion of four Ganoderma lucidum spore protein chains with four enzymes; Table S3: Results regarding the water solubility, toxicity, and allergenicity of peptide fragments with an activity score greater than 0.5.
