# Supplementary material for: Keap1-Inhibitory Peptides from Ganoderma lucidum Spores: Virtual Enzymolysis, Fragmentomics and Antioxidant Mechanism
Source: Molecules. 2026 Jun 18;31(12):2157. doi: 10.3390/molecules31122157 (PMC13304776; doi:10.3390/molecules31122157)
Supplement: Supplementary file 1 [file molecules-31-02157-s001.zip › molecules-4292401-supplementary.pdf]

Keap1-inhibitory Peptides from Ganoderma lucidum spores: Virtual Enzymolysis, Fragmentomics and Antioxidant Mechanism

Beibei Chen[1,2], Liang He[1], Qi Huang[2], Yanbin Wang[1*]

1 Key Laboratory of Biological and Chemical Utilization of Zhejiang Forest Resources, Zhejiang Academy of Forestry, Hangzhou 310023, China

2 School of Biological and Chemical Engineering, Zhejiang University of Science and Technology, Hangzhou 310023, China

*Corresponding：wangyanbin@zjforestry.ac.cn (Y.B. Wang) Tel.: +86 571 87798225; Fax: +86 571 87798206.

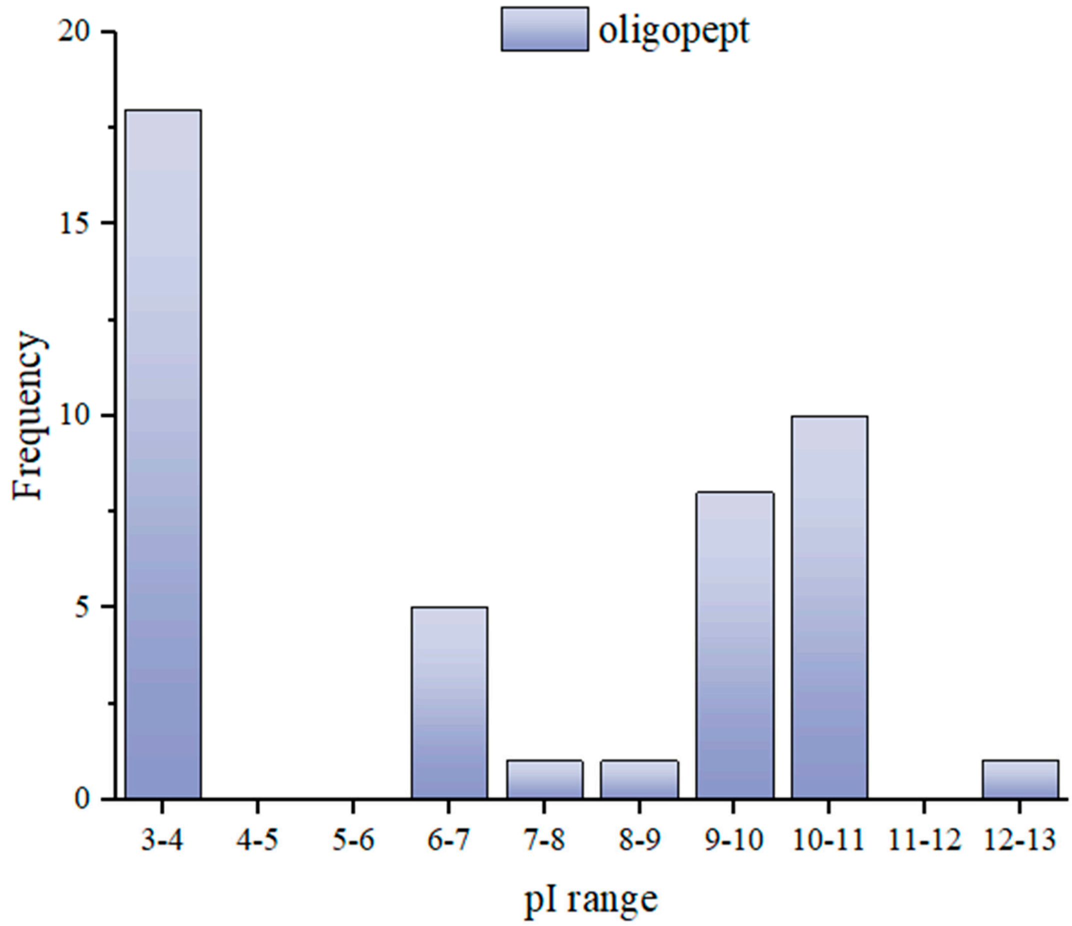

Figure S1. Isoelectric point distribution of peptides

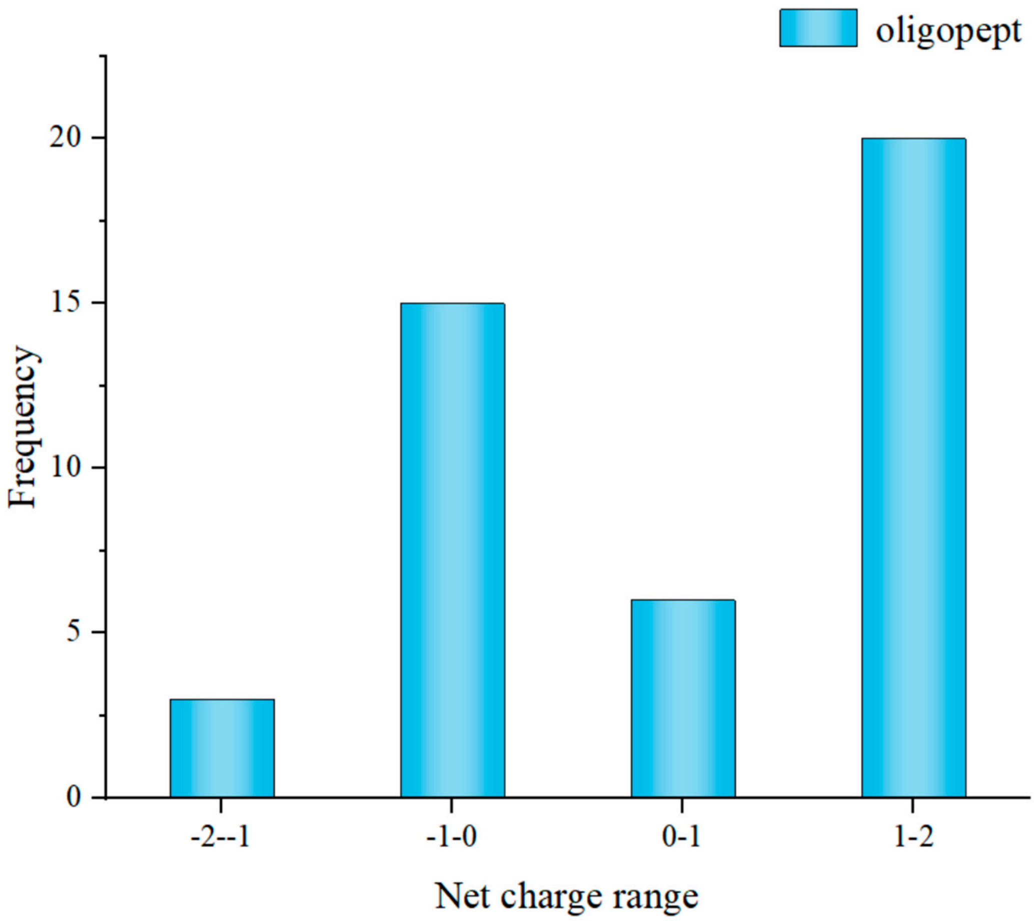

Figure S2. The Net charge of the peptide

Table S1. The basic information of four selected protein chains derived from the spore powder of Ganoderma lucidum

Uniprot ID*	The protein name	Amino acids numbers	Molecular weight
Q92429	Superoxide dismutase [Mn], mitochondrial	200	22.170 Da
B4YA15	Farnesyl pyrophosphate synthase	360	41.014 Da
G9BIY1	Diphosphomevalonate decarboxylase	400	43.302 Da
A0SJQ5	Squalene synthase	467	54.018 Da

Note: *the information of four proteins was collected from the website of www.Uniprot.org

Table S2. The peptide activity produced by the enzymatic digestion of four Ganoderma lucidum spore protein chains with four enzymes.

Peptides	Enzyme	Activity	Peptides	Enzyme	Activity	Peptides	Enzyme	Activity	Peptides	Enzyme	Activity
CF	Pepsin	0.997	IPL	Pepsin	0.674	DAF	CTRC	0.805	MY	PK	0.843
PF	Pepsin	0.993	SKF	Pepsin	0.674	QAF	CTRC	0.793	NPSF	PK	0.843
GW	Pepsin	0.993	CA	Pepsin	0.668	TRF	CTRC	0.788	NCCPI	PK	0.841
PCW	Pepsin	0.993	PIL	Pepsin	0.642	SDW	CTRC	0.773	KDPF	PK	0.840
GGF	Pepsin	0.987	DPL	Pepsin	0.642	GHSF	CTRC	0.771	RQF	PK	0.835
RF	Pepsin	0.987	HPTSGW	Pepsin	0.629	EPF	CTRC	0.757	CY	PK	0.831
RW	Pepsin	0.978	RL	Pepsin	0.626	AM	CTRC	0.745	TF	PK	0.827
DMF	Pepsin	0.966	CHY	Pepsin	0.624	THPF	CTRC	0.738	PL	PK	0.811
PPIF	Pepsin	0.954	MNY	Pepsin	0.586	ADGPL	CTRC	0.722	GL	PK	0.809
HW	Pepsin	0.953	RRL	Pepsin	0.584	AAIW	CTRC	0.719	TRF	PK	0.788
IF	Pepsin	0.949	VSKPF	Pepsin	0.575	TPNW	CTRC	0.712	DNF	PK	0.775
SF	Pepsin	0.949	RMA	Pepsin	0.574	IM	CTRC	0.697	SDW	PK	0.773
SGF	Pepsin	0.947	CRSL	Pepsin	0.568	KAF	CTRC	0.682	GHSF	PK	0.771
IW	Pepsin	0.944	PDL	Pepsin	0.567	RGL	CTRC	0.679	QGSGW	PK	0.770
DF	Pepsin	0.942	GDL	Pepsin	0.547	IPL	CTRC	0.674	EPF	PK	0.757
NF	Pepsin	0.941	RY	Pepsin	0.544	SKF	CTRC	0.674	DGPL	PK	0.748
RRF	Pepsin	0.940	PA	Pepsin	0.534	AVKPGW	CTRC	0.665	THPF	PK	0.738
NW	Pepsin	0.934	GSVDNF	Pepsin	0.534	RL	CTRC	0.626	PY	PK	0.737
NGF	Pepsin	0.932	GA	Pepsin	0.522	CHY	CTRC	0.624	MGA	PK	0.719

CCGL	Pepsin	0.924	VSW	Pepsin	0.508	DM	CTRC	0.607	TPNW	PK	0.712
GSGW	Pepsin	0.921	NDPY	Pepsin	0.503	QM	CTRC	0.607	SPDGL	PK	0.699
PSF	Pepsin	0.920	FFWPR	Tryp	0.995	EF	CTRC	0.599	MA	PK	0.693
KF	Pepsin	0.907	HFR	Tryp	0.884	VAW	CTRC	0.586	SGHSF	PK	0.693
ML	Pepsin	0.895	DFR	Tryp	0.867	DACY	CTRC	0.562	RGL	PK	0.679
RGHF	Pepsin	0.891	YWGK	Tryp	0.814	RY	CTRC	0.544	SKF	PK	0.674
MGL	Pepsin	0.885	ANGFR	Tryp	0.803	DRPTM	CTRC	0.538	CA	PK	0.668
CL	Pepsin	0.880	GR	Tryp	0.766	GSVDNF	CTRC	0.534	CI	PK	0.660
GNIGF	Pepsin	0.854	EIWGR	Tryp	0.592	CF	PK	0.996	PGGKL	PK	0.658
DPSF	Pepsin	0.846	DGGR	Tryp	0.571	GF	PK	0.995	RL	PK	0.626
MY	Pepsin	0.843	LR	Tryp	0.570	GW	PK	0.993	QDDF	PK	0.624
NPSF	Pepsin	0.843	QPILR	Tryp	0.554	GGF	PK	0.987	CHY	PK	0.624
KDPF	Pepsin	0.840	YR	Tryp	0.525	RF	PK	0.987	EF	PK	0.599
CY	Pepsin	0.831	LDACYR	Tryp	0.518	RW	PK	0.978	MNY	PK	0.586
TF	Pepsin	0.827	CF	CTRC	0.996	DMF	PK	0.966	EW	PK	0.586
VF	Pepsin	0.815	GW	CTRC	0.993	SF	PK	0.949	SSRNNF	PK	0.578
GL	Pepsin	0.809	GGF	CTRC	0.987	SGF	PK	0.947	PREHW	PK	0.577
SIF	Pepsin	0.805	RF	CTRC	0.987	DF	PK	0.942	RMA	PK	0.574
TRF	Pepsin	0.788	RW	CTRC	0.978	NF	PK	0.941	SGGGI	PK	0.573
SDW	Pepsin	0.773	AW	CTRC	0.967	KPGW	PK	0.940	CRSL	PK	0.568
GHSF	Pepsin	0.771	IF	CTRC	0.949	DGF	PK	0.939	PDL	PK	0.567
GMPQ	Pepsin	0.752	SF	CTRC	0.949	SW	PK	0.934	GRA	PK	0.559

DDF	Pepsin	0.749	RRCW	CTRC	0.944	NGF	PK	0.932	NDSF	PK	0.558
DGPL	Pepsin	0.748	DF	CTRC	0.942	QW	PK	0.929	PEDPSF	PK	0.548
THPF	Pepsin	0.738	CCGL	CTRC	0.924	CCGL	PK	0.924	PI	PK	0.547
HGTF	Pepsin	0.737	KF	CTRC	0.907	PSF	PK	0.920	RY	PK	0.544
PY	Pepsin	0.737	RGHF	CTRC	0.891	KF	PK	0.907	GQRY	PK	0.542
MGA	Pepsin	0.719	CL	CTRC	0.880	HCHGF	PK	0.898	PA	PK	0.534
TPNW	Pepsin	0.712	WPREIW	CTRC	0.868	ML	PK	0.895	DNKCSW	PK	0.531
SPDGL	Pepsin	0.699	GNIGF	CTRC	0.854	RGHF	PK	0.891	GA	PK	0.522
MA	Pepsin	0.693	KDPF	CTRC	0.840	MGL	PK	0.885	GI	PK	0.522
SGHSF	Pepsin	0.693	RQF	CTRC	0.835	CL	PK	0.880	KDGGRL	PK	0.519
SPDGL	Pepsin	0.699	CY	CTRC	0.831	RDF	PK	0.856	SGRNL	PK	0.513
GMQ	Pepsin	0.691	TF	CTRC	0.827	SKPF	PK	0.847	NDPY	PK	0.503
RGL	Pepsin	0.679	GL	CTRC	0.809	DPSF	PK	0.846			

Note: *The peptide sequences represent the amino acid sequences of peptides derived from the hydrolysis of Ganoderma lucidum spore protein by different proteases; the enzyme names indicate the types of hydrolytic enzymes used in the enzymatic hydrolysis, where Pepsin refers to pepsin, Tryp to trypsin, CTRC to chymotrypsin C, and PK to proteinase K; the activity refers to the bioactivity score of the peptides predicted by computational bioinformatics models, with scores ranging from 0 to 1, where higher scores indicate stronger predicted bioactivity of the peptide segments.

Table S3. Results regarding the water solubility, toxicity, and allergenicity of peptide fragments with an activity score greater than 0.5.

Peptide Sequence	SVM Score	Prediction	Hydrophobialty	Hydropathicity	Hydrophillicity	Moi wt
TRF	-0.77	Non-Toxin	-0.44	-0.80	0.03	422.51
SDW	0.79	Non-Toxin	0.20	-1.73	-0.03	406.42
DDF	-0.82	Non-Toxin	-0.28	-1.40	1.17	395.39
DGPL	-0.71	Non-Toxin	-0.02	0.43	0.30	400.48
SPOGL	-0.91	Non-Toxin	-0.07	-0.50	0.30	487.57
RGL	0.77	Non-Toxin	-0.36	-0.37	0.40	344.45
SKF	-0.75	Non-Toxin	-0.25	-0.63	0.27	380.47
DPL	0.79	Non-Toxin	0.09	-0.43	0.40	343.41
RL	-0.80	Non-Toxin	-0.61	-0.35	0.60	287.38
RRL	0.84	Non-Toxin	-1.00	-1.73	1.40	443.58
VSKPF	-0.95	Non-Toxin	-0.06	0.14	-0.14	576.75
RMA	-0.83	Non-Toxin	-0.42	0.27	0.40	376.50
CRSL	-0.71	Non-Toxin	0.36	0.25	0.12	477.62
PDL	-0.82	Non-Toxin	-0.09	-0.43	0.40	343.41
GDL	-0.81	Non-Toxin	-0.01	-0.03	0.40	303.35
RY	-0.80	Non-Toxin	-0.87	-2.90	0.35	337.39
GSVDNF	-0.89	Non-Toxin	-0.05	-0.20	-0.00	637.72
NDPY	-0.72	Non-Toxin	0.35	-2.48	0.23	507.54
GR	-0.79	Non-Toxin	-0.80	-2.45	1.50	231.27
EIWGR	-0.76	Non-Toxin	-0.22	-0.96	0.16	659.81
DGGR	-0.76	Non-Toxin	-0.54	-2.20	1.50	403.44
LR	0.80	Non-Toxin	-0.61	-0.35	0.60	287.38
QPILR	-1.11	Non-Toxin	-0.25	-0.26	-0.06	625.84
YR	-0.80	Non-Toxin	-0.87	-2.90	0.35	337.39
LDACYR	-0.55	Non-Toxin	-0.27	-0.20	0.07	739.91
EPF	-0.83	Non-Toxin	-0.03	-0.77	0.17	391.45
ADGPL	-0.63	Non-Toxin	0.03	0.02	0.14	471.57
KAF	-0.78	Non-Toxin	-0.08	0.23	0.00	364.47
EF	-0.80	Non-Toxin	-0.01	-0.35	0.25	294.32
DACY	-0.59	Non-Toxin	-0.10	0.12	-0.20	470.53
DRPTM	-0.73	Non-Toxin	-0.49	-1.66	0.86	616.76
DNF	0.83	Non-Toxin	-0.25	-1.40	0.23	394.41
PGGKL	0.93	Non-Toxin	-0.06	0.50	0.24	470.64
QDDF	-0.97	Non-Toxin	-0.38	1.93	0.93	523.54
EW	-0.80	Non-Toxin	-0.12	2.20	-0.20	333.36
SSRNNF	-0.86	Non-Toxin	-0.49	1.72	0.25	723.81
PREHW	-0.70	Non-Toxin	-0.50	2.74	0.42	723.85
GRA	-0.79	Non-Toxin	-0.45	-1.03	0.83	302.38

NDSF	-0.82	Non-Toxin	-0.25	-1.25	0.25	481.50
PEDPSF	-0.83	Non-Toxin	-0.19	-1.37	0.63	690.77
GQRY	-0.74	Non-Toxin	-0.57	-2.43	0.23	522.61
DNKCSW	0.42	Non-Toxin	-0.38	-1.68	0.35	751.88
KDGGRL	-0.70	Non-Toxin	-0.46	-1.48	1.20	644.81
SGRNL	-0.57	Non-Toxin	0.39	-1.08	0.34	545.66

Note: *The SVM Score is the toxicity prediction score derived from the support vector machine model and serves as the core evaluation metric for peptide toxicity potential. The Prediction is the toxicity outcome based on the SVM Score, where "Non-Toxin" indicates the peptide is predicted to be non-toxic. Hydrophobicity is the hydrophobicity index, with negative values indicating a hydrophilic tendency and positive values indicating a hydrophobic tendency. Hydropathicity is the overall hydrophilicity/hydrophobicity index of the peptide, where negative values indicate hydrophilicity and positive values indicate hydrophobicity. Hydrophilicity is the hydrophilicity index, with larger values indicating stronger hydrophilicity of the peptide. Mol wt is the relative molecular mass of the peptide.
